# Photodynamic therapy for synovial hyperplasia in patients with refractory rheumatoid arthritis: a study protocol for a randomized, double-blind, blank-controlled prospective trial

**DOI:** 10.1186/s13063-021-05640-8

**Published:** 2021-10-09

**Authors:** Xiaofeng Zhao, Fangfang Zuo, Ensheng Chen, Yanan Bi, Yanyan Cao, Yi Yuan, Kaiqin Li, Yanan Xuan, Libo Li, Lijuan Wan, Xiangqun Zhang, Feifei Yan, Jiyuan Zhou, Kun Yin, Changhong Xiao

**Affiliations:** 1 Integrated Hospital of Traditional Chinese Medicine, Southern Medical University, Guangzhou, China; 2 Department of Biostatistics, School of Public Health, Southern Medical University, Guangzhou, China; 3 General Outpatient Clinic of Chigang Street Community Health Service Centre, Guangzhou, China

## Abstract

**Background:**

Persistent synovial hyperplasia with inflammation in rheumatoid arthritis is one of the main pathogeneses of refractory rheumatoid arthritis (RRA). Photodynamic therapy (PDT) causes less trauma than steroid injections or arthroscopic synovectomy while providing stronger targeting and more durable curative effects. The aim of this trial was to evaluate the short-, medium-, and long-term clinical efficacy of PDT when applied as a treatment for RRA synovial hyperplasia and synovitis.

**Methods and analysis:**

This protocol is for a single-center, randomized, double-blind, blank-controlled prospective trial. A sample of 126 RRA patients will be randomly divided into 3 groups: the control group, the “PDT once” group, and the “PDT twice” group, with 42 participants per group. The trial will be conducted by the Rheumatology and Immunology Department of the Integrated Hospital of Traditional Chinese Medicine, Southern Medical University. The Ultrasound Compound Score of Synovitis (UCSS) has been selected as the primary outcome measure. The secondary outcome measures include knee joint clinical assessments, ratio of relapse, duration of remission, Disease Activity Score in 28 joints (DAS28), inflammation indexes, serum concentrations of specific antibodies, and changes in articular structures as detected by X-ray scans in the 48th week. The improvement ratios of the UCSS at the 8th, 24th, and 48th weeks (compared with baseline) reflect short-, medium-, and long-term time frames, respectively.

**Ethics and dissemination:**

The protocol was approved by the Medical Ethics Committee of the Integrated Hospital of Traditional Chinese Medicine, Southern Medical University, China (Approval No. granted by the ethics committee: NFZXYEC-2017-005) and then entered in the Chinese Clinical Trials Registry under registration number ChiCTR1800014918 (approval date: February 21, 2018). All procedures are in accordance with Chinese laws and regulations and with the Declaration of Helsinki by the World Medical Association (WMA). Any modifications of this protocol during execution will need additional approval from the Ethics Committee of our hospital.

**Trial registration number:**

ChiCTR1800014918.

## Background

Rheumatoid arthritis (RA) is a chronic inflammatory disease characterized by persistent joint inflammation and synovial hyperplasia. Without appropriate treatment, patients with RA suffer from joint deformities and progressive functional impairment. Owing to the implementation of the treat-to-target strategy and the availability of biologic therapies, the prognosis for patients with RA has significantly improved [1]. However, some patients with RA respond poorly to multiple drugs (first-line drugs have a tolerance rate of more than 20%, and the effectiveness rate of many biological agents is only 70%) [2]. It is a common phenomenon in ordinary clinical practice for swelling and pain (due to synovitis, including synovial hyperplasia and synovial inflammation) to persist or recurrently relapse in some joints (such as the elbow, knee, and ankle) of RA patients with a low level of main systemic inflammation and arthritis. The persistence of synovitis results in the erosion of cartilage and bone, which further degrades the response to treatment. In the clinic, this type of RA is known as refractory RA (RRA). Aside from conventional medicines that act on the whole body, local injection with hormones in the joint cavity and arthroscopic synovectomy are among the most common methods of RRA therapy, providing short-term relief. However, repeated local injections increase the tendency toward infection and crystallization, causing poor compliance [3]. Arthroscopic synovectomy can also relieve the symptoms temporarily, but patients usually relapse within half a year, sometimes even progressing to a more serious condition because of incomplete resection and the inability to reverse synovial pathology [4]. Therefore, a long-acting therapy administered locally to the joints is urgently needed in the clinic.

Photodynamic therapy (PDT) involves activating a photosensitizer (PS) with a specific wavelength, generating photochemical reactions that are toxic to adjacent tissues [5]. Additionally, utilizing the affinity of PS to hypermetabolic tissues and cells, overproliferated inflammatory cells can be eliminated selectively by the ROS from photoactivated PS molecules, which is a significant advantage of PDT [6].

The goal of applying PDT in the treatment of RRA is to modify the characteristic local pathology-synoviocyte proliferation and inflammatory cell infiltration-through specific PS retention in synoviocytes and inflammatory cells, enabling the selective suppression of synoviocyte proliferation and inflammatory cell infiltration without damage to the normal tissues. PDT has good prospects because of its targeted delivery, high effectiveness, and relatively mild adverse effects; other therapies lack these advantages.

PDT has been used clinically for more than 20 years to treat tumors. Although PDT has shown significant effects in an experimental collagen-induced arthritis (CIA) animal model [7], its effectiveness in the clinical treatment of RA has not been reported until now. To explore the effects of PDT in relieving local synovial proliferation in the knee joints of patients with RRA, we intend to carry out this prospective trial as a pilot study.

## Methods

### Trial design

**Fig. 1:**
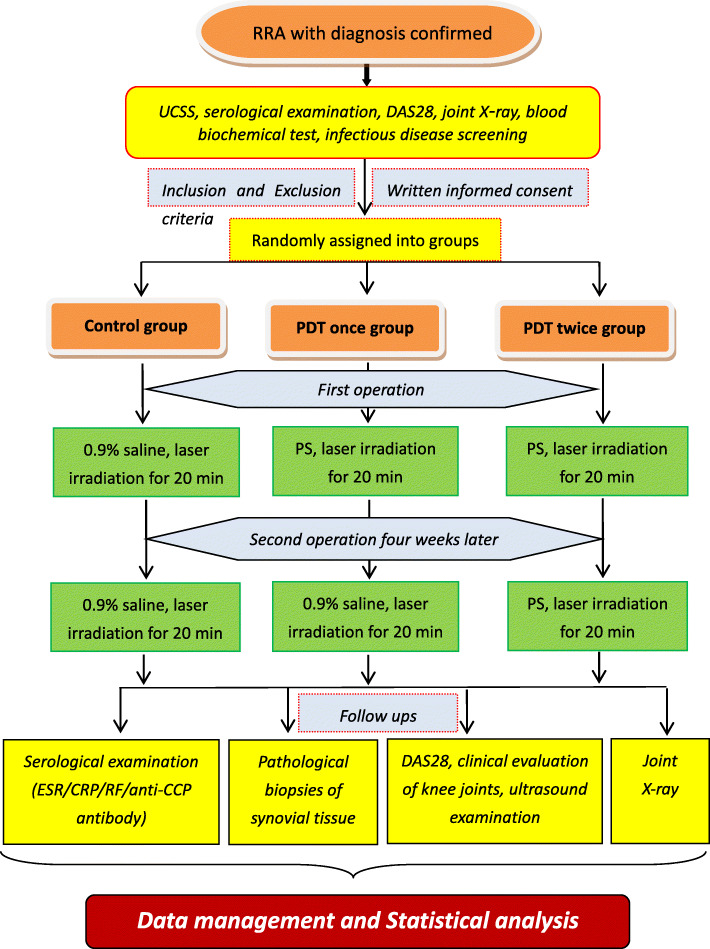
Trial flow diagram

This is a single-center, randomized, double-blind, blank-controlled trial with 3 groups: the control group, the “PDT once” group, and the “PDT twice” group. The curative effect will be compared statistically across groups. There will be 126 participants in total, with 42 in each group. The trial will be conducted at the Rheumatology and Immunology Department at the Integrated Hospital of Traditional Chinese Medicine, Southern Medical University. Further details can be seen in the flowchart of the trial design in Fig. 1.

This study will be carried out according to the Standard Protocol Items: Recommendations for Interventional Trials (SPIRIT) statement, and a SPIRIT checklist has been completed.

### Patients

#### RRA diagnostic criteria

(1) The 1987 American Rheumatism Association (ARA) classification criteria for RA are as follows: (i) morning stiffness in and around joints lasting at least 1 hour before maximal improvement; (ii) Soft tissue swelling or arthritis of 3 or more joints observed by a physician; (iii) Swelling or arthritis of the proximal interphalangeal, metacarpophalangeal, or wrist joints; (iv) Symmetric swelling (arthritis); (v) Rheumatoid nodules; (vi) The presence of rheumatoid factor; and (vii) radiographic erosions and/or periarticular osteopenia in hand and/or wrist joints. Criteria i to iv must have existed for at least 6 weeks. Rheumatoid arthritis is defined as the presence of 4 or more criteria [8]. (2) RA will be classified as RRA if low activity (Disease Activity Score in 28 joints calculated with erythrocyte sedimentation rate (DAS28-ESR)<3.2) or remission (DAS28-ESR<2.6) [9, 10] is not achieved in response to more than 3 months of regular, sufficient treatment, including traditional drugs such as nonsteroidal anti-inflammatory drugs, more than two DMARDs and/or biologic DMARDs, or combined therapy.

#### The inclusion and exclusion criteria

The inclusion and exclusion criteria for the patients are presented in Table 1.

**Table 1:** Inclusion and exclusion criteria

*Inclusion criteria*	
*1. Male or female patients aged between 18 and 70 years* *2. Patients who meet the diagnostic criteria for RRA* *3. Patients with swelling of at least one knee for 1 month* *4. Patients with synovial hyperplasia confirmed by ultrasound or MRI: UCSS ≥ 2 points and/or single-site MR scores ≥2 points* *5. Patients who are capable of signing informed consent and completing the trial as required* *6. Patients who have good compliance and will complete follow-up*	
*Exclusion criteria*	
*1. Patients with severe primary diseases, such as disorders of the cardiovascular system, liver, kidneys, brain, or hematopoietic system* *2. Patients with infections or coagulation abnormalities* *3. Breastfeeding or pregnant women* *4. Patients who are allergic to anesthetics or PSs* *5. Patients who have undergone intra-articular drugs injection within the past month* *6. Patients with arthritic knee joints that show osteotropic ankylosis on X-ray examination*	

### Groups and sample size

The necessary sample size was estimated to be 99 based on a preliminary trial with a small sample size. During the preliminary phase, eighteen participants were included and randomly divided into 3 groups of 6. According to the protocol, differences in UCSS between week 8 and the baseline value were detected. The mean ± standard deviation of UCSS reduction was obtained at week 8 and week 0 individually: 0.33±0.817, −0.80±0.837, −1.0±1.000. The standard deviation was 0.873, and PASS 11 software was used to estimate the sample size by means of the following formula:

$$ 1-\beta =1- probF\;\left[{F}_{1-a,G-1,N-G,}\;\left(G-1\right),N-G, NV/{\sigma}^2\right] $$

The significance level, *α*, was set to 0.05; the power of a test, 1 − *β*, was 0.80; the number of groups, *G*, was 3; *N* represents the sample size; *V* represents the variance of the group averages, $V=\Sigma \left({\left({\mu}_i-\overline{\mu}\right)}^2\right)/G$; σ represents the standard deviation.

A total of 126 participants will be included to allow for 20% loss to follow-up. There will be 3 groups:

1. Control group: (*n*=42) These individuals will receive micro-arthroscopic surgical procedures twice (day 0 and 4th week), and 0.9% saline will be administered instead of PS.
2. PDT once group: (*n*= 42) Participants will receive PDT on day 0, followed by 0.9% saline administration instead of PS in the 4th week.
3. PDT twice group: (*n*=42) Individuals will receive PDT twice: once on day 0 and once in the 4th week.

### Randomization and blinding

We will recruit participants from our hospital, clinic, and by referral from other hospitals. To participate, all candidates must sign an informed consent form before entering the screening period. After the baseline survey, all identified participants will be randomly assigned to the 3 groups as previously stated. The block randomization method will be used for 1:1:1 randomization to ensure a balanced distribution between 3 groups. The random numbers will be generated using SAS 9.12 software. On this basis, randomization will be conducted by a professional nurse at the hospital follow-up center.

The researchers responsible for evaluations, application of the treatment protocol, and statistical analysis will be blinded to the group allocation of participants. The participants will also be blinded to group allocation. All researchers and patients in this trial will be informed not to communicate regarding the details of the groups. There is no difference in the appearance of PS solution and saline, enabling the double blinding to be maintained. PS or saline will be prepared by the professional nurse at the follow-up center, according to the random grouping table. Participants will be unblinded at the termination of this trial.

If emergency unblinding is deemed necessary, for example, if there is a serious adverse event (SAE) such as instrument fracture in the articular cavity, tissue hematoma, infection, or structural damage, researchers should determine the relevance of the event based on the grouping information. Effective treatment will be administered to those participants.

### Intervention

All patients will receive steroids, nonsteroidal anti-inflammatory drugs, and/or DMARDs (e.g., conventional synthetic DMARDs, biologic DMARDs, and targeted synthetic DMARDs) in accordance with the treatment guidelines.

**Fig. 2:**
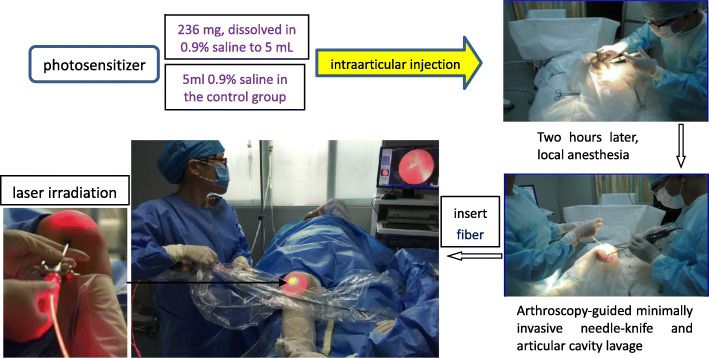
Photodynamic therapy process and scenarios

Participants in the PDT treatment groups will be injected with a PS (aminolevulinic acid, 236 mg, dissolved in 0.9% saline to 5 mL) in the articular cavity of the knee before the operation. Participants in the control group will be injected with 5 mL of 0.9% saline instead of PS. Local anesthesia will be performed 2 h later after joint injection. Then, arthroscopy-guided minimally invasive needle-knife arthroscopy for single-hole exploration and articular cavity lavage will be performed. Pathological biopsies of the synovial tissue will be taken before laser irradiation. A laser optical fiber will be inserted into the articular cavity through a cannula. Then, with the knee joint half bent, three sites will receive laser irradiation for a total of 20 minutes: the posterior patella for 10 min and the medial and lateral condyles of the femur for 5 min each. The following parameters will be used for the laser: wavelength, 630 nm; power, 150 mW. A second operation will be performed at the 4th week. Further details are shown in Fig. 2.

### Evaluation procedure

The researchers will record participants’ postoperative improvement and general condition, including body temperature and respiration, pulse, and heart rate, during face-to-face follow-up visits. The items evaluated during each follow-up visit are shown in Table 2. All the data will be uploaded to the case report form (CRF) by rigorously trained data collectors. To reduce the rate of loss to follow-up, we will also communicate with participants by a variety of methods, including WeChat, telephone, text message, and e-mail, to remind them to return for further examination.

**Table 2:** Follow-up plan

***Follow-up time***	***Evaluation index***
*Baseline*	*UCSS, clinical evaluation of knee joints, DAS28, antibody levels, inflammation indicators, X-ray, routine blood test, routine urine test, blood biochemical test, infectious disease screening*
*W0* *(first operation)*	*Pathological biopsies of synovial tissue*
*W4±3 d* *(second operation)*	*UCSS, clinical evaluation of knee joints, DAS28, antibody levels, inflammation indicators, pathological biopsies of synovial tissue*
*W8±3 d*	*UCSS, clinical evaluation of knee joints, DAS28, antibody levels, inflammation indicators, blood biochemical test*
*W24±5 d*	*UCSS, clinical evaluation of knee joints, DAS28, antibody levels, inflammation indicators*
*W48±5 d*	*UCSS, clinical evaluation of knee joints, DAS28, antibody levels, inflammation indicators, X-ray*

*W* week, *d* days

### Outcome and safety measures

#### Outcome measures

1. Primary outcome measure

The Ultrasound Compound Score of Synovitis (UCSS) will be calculated according to the EULAR-OMERACT combined scoring system for grading synovitis in rheumatoid arthritis [11]. Improvement in the UCSS compared with the control group will be the primary endpoint.

(2) Secondary outcome measures
  1. Clinical evaluation of knee joints: The circumference will be measured 2 cm above the upper edge of the patella, and joint flexion and extension will be measured to assess the range of motion.
  2. DAS28-ESR: DAS28-ESR= 0.56 × the total number of tender joints + 0.28 × the total number of swollen joints + 0.70 × ln(ESR) + 0.014 × general health assessment score.
  3. Antibody levels: Serum will be collected by centrifugation, and then the RF and anti-CCP antibodies will be detected by a standard commercially available test kit.
  4. Inflammation indicators: Serum will be obtained by centrifugation, and then ESR and CRP will be detected by standard commercially available test kits.
  5. X-ray: X-rays of the knee joint will be used to evaluate bone destruction.
  6. Pathological biopsies of synovial tissue: The synovium of the joint, which will be acquired before laser irradiation during each operation, will be fixed, embedded by dehydration, and sectioned. The synovium will be stained with hematoxylin and eosin for histological evaluation, and synovial histopathology will be observed, including synovial cell proliferation, inflammatory cell infiltration, and angiogenesis (pannus formation). The pathological biopsy of synovial tissue will strictly follow the clinical diagnostic protocol. The research nurses will collect specimens with special specimen boxes, record basic information on the subjects, and then send specimens to the pathology department for testing and preservation as soon as possible.

#### Safety measures

1. General check-up: Liver function and renal function will be examined to evaluate the effect of basic drug therapy.
2. Special check-up: After knee joint treatment, wound healing should be observed, and routine blood examination of white blood cells will be performed to assess whether there is an infection.

The study will record and process any PDT-related adverse events or SAEs experienced by participants.

### Data management and statistical analysis

#### Data management

The project administrator will develop strict procedures to ensure that the data are well preserved and traceable. During this trial, the raw data from participants and clinical assessments will be collected by data collectors on the CRFs created by the statistician. Furthermore, all of the data will be precisely entered into a password-protected computer within 2 weeks after data collection. After data collectors have entered all the data, the CRFs and signed informed consent forms will be handed over to the administrator for preservation.

The quality control personnel and inspector, as the designated representatives of the principal investigator, will have the right to supervise this trial and determine whether it is being carried out in accordance with the trial procedures. The CRFs will be filled out by the researcher, and the data will be recorded completely, accurately, clearly, and in a timely manner. The inspector shall check the CRFs in accordance with the original data. If there are any errors or omissions, the researcher will be asked to correct them in a timely manner and to sign and date the corrections in order to keep the original record in an easily accessible form. The CRFs will be handed over to the clinical trial data manager for data entry and management. For completed case report forms, there will be corresponding records and signatures by the researchers, inspectors and administrators. All these records will be properly preserved. To ensure confidentiality, the data disseminated to all project team members will be free of any information that identifies the participants.

The Department of Health Statistics of Southern Medical University will be responsible for data entry and modification. The data administrators need to check the CRFs again before entering the data. The researchers should answer any questions from the question request form (QRF) and return to the clinical inspector, who should properly preserve the document, as soon as possible. The data manager will record two copies of the data and verify that they are identical. During the data entry process, if errors are found in the entries, they should be registered and reported in a timely manner with the input of the leading researchers. A data range check and a logical check will be performed based on the ranges and interrelations of all index values in the CRF, and a corresponding computer program will be written to ensure that incorrect data cannot be entered. When a problem is found, QRFs can be issued again if necessary. After the data entry and verification have been completed as required, the original CRFs will be archived and stored in form number order and reviewed in the search catalog. The electronic data files include a database, checking procedures, analysis procedures, and analysis results, among other items. All original documents should be kept in accordance with Good Clinical Practice (GCP).

#### Data monitoring

The overall data quality will be monitored by the clinical research assistant of the Hospital GCP Center, which is independent of the sponsor and competing interests, throughout the study. The completeness and authenticity of the original data will be subject to checks at any time. After this trial is completed, all data will be kept for 15 years and then deleted.

#### Statistical analysis

SPSS 20.0 statistical software will be used to analyze the measurement data, described as the means ± standard deviations ($\overline{x}\pm s$), and the main evaluation index (improvement in the UCSS compared with the baseline value) will be analyzed by single-factor variance analysis among the three groups. ANOVA will be used for repeated measurement data of the secondary evaluation indicators to explore the principal and interactive effects of grouping and time. Multiple paired *t*-tests comparing two time points (before and after treatment) will be used to analyze repeated measurements. Single-factor ANOVA will be used to compare the differences at the same time points after treatment between the three groups. Differences will be statistically significant at *P* <0.05.

### Quality control

To ensure that the study is conducted in a consistent, scientific, and effective manner, the operators, data collectors, data analysts, and data managers will receive strict and specific training before the implementation of the clinical protocol. The operators, who are essential for ensuring the reliability of the trial, will be required to improve their theoretical and practical knowledge of PDT. In addition, to put the subjects at ease during the study, the operators will attempt to prevent any negative reactions on the part of subjects by taking some lessons on communication methods and skills. Data collectors will enter the original data into the CRF in a timely, consistent, and accurate way.

After the successful group allocation of the first subject, half of subjects, and all subjects, the Research Group and GCP Agency will send quality controllers to monitor the quality of this project in its early, middle and final stages and strictly evaluate whether the procedures were carried out in accordance with the trial protocol. The project team will organize all the members to hold an annual summary and training meeting. Our hospital and the science and technology departments of the university will also conduct an annual project assessment and invite expert groups to evaluate the progress of the implementation. The Medical Ethics Committee of the Hospital will conduct an annual follow-up review to ensure that the rights and interests of the participants are protected.

## Discussion

Traditional drugs, including nonsteroidal anti-inflammatory drugs, steroids, and disease-modifying antirheumatic drugs (DMARDs), have rapidly developed in the past 10 years and can control the general condition of most RA patients. However, the curative effects are poor in some RA patients, which reduces their quality of life and greatly increases the difficulty of RA treatment. Although refractory joints can be treated with local steroid injections or arthroscopic synovectomy, these treatments have substantial shortcomings, and the pain may not be fully resolved. As a potential therapy for the treatment of synovial hyperplasia in RRA, PDT causes less trauma than steroid injections or synovectomy while providing stronger targeting, fewer side effects, and more accurate curative effects. Patients tend to tolerate PDT treatment better than these alternatives. PDT has prospects for broad application and has demonstrated promising effects on synovitis, but there remain some problems to be solved.

We have determined that refractory synovitis is essential in RRA, and early intervention in long-term RA can be useful for reducing the proliferation and migration of synoviocytes and inhibiting inflammatory cell infiltration. The cytotoxic and immunoreactive effects of PDT can inhibit synovial hyperplasia and pannus formation, thereby reducing cartilage and bone destruction, which is critical for RRA treatment. In clinical practice, we found that the irradiation dose and the duration of treatment and scope of PDT are difficult to establish, and single therapy cannot completely eliminate synovial hyperplasia. The curative effect requires long-term clinical experience to be defined.

To date, almost all studies on the possibility of PDT to treat RA have consisted of basic research and experiments in animal models of arthritis, which have confirmed the effect of PDT. There is a lack of research on clinical efficacy evaluation until now. In view of this, this study aims to clarify the short- and long-term clinical efficacy of PDT in the treatment of RA synovitis, explore the frequency and duration of treatment, and finally form a practical standard therapy for RRA synovial hyperplasia.

We believe that with an increasing number of basic research and clinical trials, the long-term efficacy and technical problems of PDT in the treatment of RRA synovial hyperplasia will be addressed and PDT will be widely applied.

### Strengths and limitations of this study

1. The aim of this trial is to evaluate the short-, medium-, and long-term clinical efficacy of PDT in the treatment of RRA synovial hyperplasia and synovitis with a 1-year follow-up.
2. An improvement in the UCSS value compared with the baseline will be the primary endpoint based on the EULAR-OMERACT combined scoring system for grading synovitis in RRA.
3. PDT has the advantages of minimal trauma, stronger targeting, fewer side effects, and definite curative effects, which makes it more acceptable to patients than steroid injections or arthroscopic synovectomy.
4. The trial will take place at only a single center.

## Data Availability

The principal investigator, Changhong Xiao, Professor, PhD, will have access to the final trial dataset. The raw data will be shared 1 year after the end of the trial through sources such as the Clinical Trial Public Management Platform ResMan (www.medresman.org) or Chinese Rheumatism Data Center (CRDC).

## Abbreviations

RRA: Refractory rheumatoid arthritis
PDT: Photodynamic therapy
UCSS: Ultrasound compound score of synovitis
DAS28: Disease activity score in 28 joints
EULAR-OMERACT: European league against rheumatism–outcome measures in rheumatology
PS: Photosensitizer
ROS: Reactive oxygen species
ARA: American rheumatism association
DMARDs: Disease-modifying antirheumatic drugs
csDMARDs: Conventional synthetic DMARDs
bDMARDs: Biologic DMARDs
tsDMARDs: Targeted synthetic DMARDs
SAE: Serious adverse events
CRF: Case report form
ESR: Erythrocyte sedimentation rate
CCP: Cyclic citrullinated peptide
CRP: C-reactive protein
QRF: Question request form
GCP: Good clinical practice
ANOVA: Analysis of variance
CRDC: Chinese rheumatism data center
